# A Testbed for the Development and Validation of Contactless Vital Signs Monitoring Systems

**DOI:** 10.3390/s26041092

**Published:** 2026-02-07

**Authors:** Zaid Farooq Pitafi, He Yang, Jiayu Chen, Yingjian Song, Jin Ye, Zion Tse, Kenan Song, WenZhan Song

**Affiliations:** 1 School of Electrical & Computer Engineering, University of Georgia, Athens, GA 30602, USA; heyang95@uga.edu (H.Y.); jiayu.chen@uga.edu (J.C.); yingjian.song@uga.edu (Y.S.); jin.ye@uga.edu (J.Y.); kenan.song@uga.edu (K.S.); wsong@uga.edu (W.S.); 2 School of Engineering and Materials Science, Queen Mary University of London, 327 Mile End Rd, Bethnal Green, London E1 4NS, UK; z.tse@qmul.ac.uk

**Keywords:** cardiorespiratory testbed, contactless vital signs monitoring, signal processing, system development, algorithm validation

## Abstract

Contactless monitoring of vital signs such as heart rate (HR) and respiratory rate (RR) has gained significant attention, with vibration-based sensors like geophones showing promise for accurate, non-invasive monitoring. However, most existing systems are developed with healthy subjects and may not generalize well to extreme physiological ranges, such as those observed in infants or patients with arrhythmia. Moreover, the underlying mechanisms of cardiorespiratory vibration dynamics remain insufficiently understood, limiting clinical adoption of these systems. To address these challenges, we present a programmable cardiorespiratory testbed capable of generating realistic HR and RR signals across a wide range (HR: 40–240 bpm, RR: 8–40 bpm). Our system uses a voice coil motor that acts as the vibration source, driven by a Raspberry Pi-based control circuit. Unlike similar systems that use separate modules for heart and lung signals, our setup generates both signals using a single motor. The synthetic signals exhibit a strong correlation of 0.85 compared with data from 75 human subjects. We use this system to design signal processing-based algorithms for vital signs monitoring and demonstrate their robustness for extreme physiological ranges. The proposed system enhances the understanding of cardiorespiratory vibration dynamics while significantly reducing the time and effort required to collect real-world data.

## 1. Introduction

Monitoring vital signs such as heart rate (HR) and respiratory rate (RR) provides critical insight into an individual’s health. These are considered primary vital signs that aid in detecting potential medical conditions. Some of these conditions include irregular heartbeats such as Tachycardia (increased HR) that may be caused by heart disorders, and Bradycardia (low HR) that could be caused by advanced heart diseases. Similarly, monitoring RR is important for diagnosing conditions such as sleep apnea [1], pneumonia, heart failure, metabolic imbalances, and cardiac arrest. The traditional monitoring methods such as ECG monitors, which are considered the gold standard, are not practical for extended use because of patient discomfort. Recent advances in technology have made it possible to have continuous monitoring of these vital signs by using non-invasive techniques. These systems can help track vital signs and provide real-time data that is invaluable in both home and clinical settings. Some of these techniques rely on using wearable devices [2,3]. However, the users feel a certain discomfort or in some cases, do not prefer to wear them to sleep. Further, some elderly users may forget to charge their devices, which puts their health monitoring at significant risk.

To address these issues, contactless solutions have been gaining popularity recently. They use various sensing technologies such as infrared thermography, radar, and optical sensors to estimate vital signs. However, these technologies pose privacy concerns for users. Therefore, contactless sensors deployed under the bed that use the cardiac vibration for vital signs monitoring have been proposed as an alternative. These systems use ballistocardiography [4] or geophone sensors [5,6,7] to estimate vital signs with high accuracy. The vibration sensor is placed under the bed and detects mini-vibrations caused by the movement of heart and chest contractions.

However, the use of vibration-based sensors is a complex technique. The dynamics of the cardiorespiratory vibrations have not been explored in detail. Further, this technology is extremely sensitive to noise and can pick up any small vibration coming from the background due to human activity or other sources (electrical appliances etc.). Therefore, the extraction of cardiac signal and relevant features for vital signs estimation from the raw data is an extremely challenging process. Further, the clinical validation of such systems requires extensive testing and validation for extreme cases. The collection of real-world data for development and optimization of these systems is not straightforward due to data acquisition, security and integrity concerns, especially for certain subjects such as infants. Also, this emergent technology does not have any public datasets available, compared to the existing technologies. Therefore, having a testbed to study the cardiorespiratory dynamics and perform extensive evaluations would enable the development and validation of these systems, including extreme cases that may not be obtained in real collected datasets. This is especially significant for the detection of heart and lung abnormalities such as Atrial Fibrillation (AFib).

**Figure 1:**
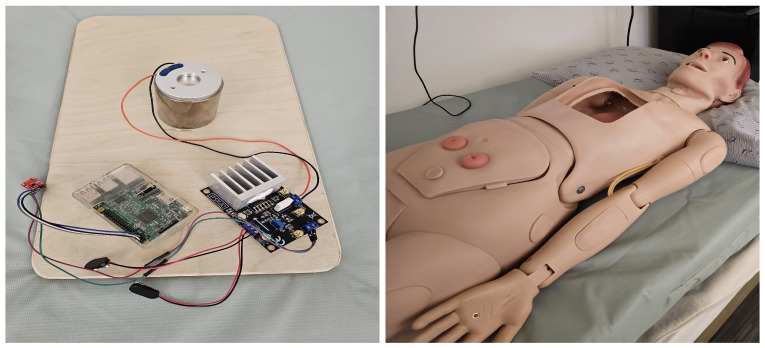
Testbed setup.

In this paper, we present SimDot, a cardiorespiratory testbed that can be used for development, testing and validation of contactless vital signs monitoring systems that use geophone-based sensors. Our testbed uses a voice coil motor as the cardiac vibration source. The motor is controlled by a Raspberry Pi with the help of a Digital to Analog (DAC) converter and a Power Amplifier. To collect the vibrations generated by this motor, a vibration-based sensor (geophone) is placed under the bed that can sense the vibrations generated due to heart and respiratory beats. The results show that our system generates a highly accurate signal, presenting strong correlation (0.85) with a large human dataset (75 people). The system is used to refine our existing vital signs monitoring algorithms that have been developed using the same human dataset, by increasing their robustness for higher ranges and extreme cases, with performance metrics showing Mean Absolute Error (MAE) less than 2 bpm for HR and RR. SimDot is low-cost and can be easily deployed in different environments. The proposed system is shown in Figure 1.

The major contributions of our study are summarized below:

1. We propose a cardiorespiratory testbed for development, testing and validation of geophone-based contactless vital signs monitoring systems.
2. We use a single motor setup to generate heart and lung signals compared to the similar works that use separate modules.
3. Our system facilitates validation of HR from 40 to 240 and RR from 8 to 40 beats per minute (bpm), and can be used to generate abnormal heartbeat data. The generated signal is highly accurate, showing strong correlation with real data (>0.85).
4. The testbed is used to design and improve vital signs algorithms by increasing their robustness and making them work for these extreme ranges (compared to 120 bpm HR and 25 bpm RR previously).

The remainder of this article is organized as follows. Section 2 presents the related works, covering vibration-based vital signs monitoring systems and cardiac simulator testbeds. In Section 3, we present the material and methods that include the system design and vital signs monitoring algorithms. In Section 4, we present the experimental setup and results, followed by conclusions in Section 5.

## 2. Related Works

This section is divided into two parts. In the first part, we present a brief history of advances in vibration-based contactless vital signs monitoring systems. This is followed by a study of mechanical cardiac testbeds.

### 2.1. Vibration-Based Contactless Vital Signs Monitoring Systems

In recent years, contactless systems have gained more popularity due to their ease-of-use. These systems use vibration-based sensors for estimation of vital signs. Some of the techniques rely on ballistocardiography (BCG), seismocardiography (SCG) or geophone sensors for the monitoring of vital signs. The BCG-based systems record the vibration/movements of the heart as it pumps blood to shift the body’s center of gravity during the heart beating cycle. They consist primarily of accelerometer-based approaches. The sensor is usually placed on the body or has some mechanical contact with the body (under a bed or a chair).

Starr et al. [8] performed the first study on body trembling caused by heart contraction. They constructed a mechanical table with a steel spring opposing its lateral motion. Afterwards, Mandelbaum et al. [9] performed a study that showed that improvements in BCG signal consistency over time were indicative of a recovering heart. In a later study, Starr et al. [10] observed that those subjects with hearts contracted with little force at the initial test later suffered from death and cardiac disability, chiefly coronary heart disease, in far greater numbers than those whose hearts contracted strongly. Several systems building on the same principle [11,12] have been developed since and enable the vital signs monitoring with high accuracy.

The seismocardiography (SCG)-based systems measure the vibration due to pressure within the body. They build on the same principles introduced by Starr et al. [8]. Compared to the BCG-based approaches that study the measurement of whole-body recoil forces in response to cardiac ejection, the SCG-based systems observe the local chest surface measurement of cardiac-induced vibrations. These signals are caused by cardiac mechanical processes such as valve movement, blood flow, and muscle contraction. These systems are usually placed near the sternum of body. SCG signals are recorded using accelerometers placed at a single location on the chest wall. In recent years, several systems [13,14], have been developed that show the use of SCG for vital signs monitoring. However, the relationship between SCG waves and cardiac activity has not been fully understood.

Previous works have shown the use of the geophone sensor to accurately estimate vital signs. In these systems, the geophone sensor is placed under the bed or mattress and detects the heart vibration data. Jia et al. [5] were one of the first to introduce such systems. In their study, they used Auto-Correlation Function (ACF) of the signal for HR estimation. They also showed that their system can estimate RR [15] in a subsequent work. Clemente et al. [16] showed that their system can estimate HR and RR by using a geophone sensor. Song et al. [6] showed their system can achieve high accuracy for HR and RR estimation. However, similar to the SCG-based systems, the relation between the cardiac activity and geophone-based systems has not been fully understood. Further, these systems are developed using regular human data and may not generalize well for extreme cases/abnormalities. Therefore, we propose a simulator testbed to address these issues.

### 2.2. Mechanical Simulator Testbeds

The use of vital signs monitoring for clinical purposes requires extensive validation, especially for extreme cases/abnormalities that cannot be obtained during real-world data collection. Safitri et al. [17] introduced a vital signs simulator capable of simulating ECG and Non-Invasive Blood Pressure (NIBP) parameters in a single device by using an Arduino board for control signal. This system operates on a 220V AC input voltage. They showed their system can achieve good accuracy with some errors at low HR. Their designed device was evaluated across various settings, including heart rates of 30, 60, 120, and 180 beats per minute (bpm). Narvaez et al. [18] presented a vital signs simulator for calibration of medical equipments. Their system is able to simulate ECG pulse rate and waveform by using an Arduino microcontroller. Their system was tested at three different frequencies (60 bpm, 90 bpm and 120 bpm) and achieved good accuracy.

Setiawan et al. [19] developed a system for implementation of heart rate using AD8232 and Arduino microcontrollers. Their research focused on enhancing the accuracy and efficiency of ECG data capture, contributing to advanced cardiac monitoring systems. Their system uses three essential ECG sensors interface with the AD8232, facilitating the acquisition of valuable analog ECG data. Ichimura et al. [20] developed a simulator that enables medical trainees to measure heart rate and arterial oxygen saturation by pulse oximeter (SpO2), which is generated according to a mathematical cardiorespiratory model in response to resuscitation procedures.

Kadambi et al. [21] introduced one of the first simulators for vibration-based systems. Their system proposes ballistic simulator for the BCG signals that uses separate motors to generate HR and RR signals. The heart module consists of a vibration motor which acts as an out-of-balance centrifuge, creating a vibration that is similar to the heartbeat. The lung module consists of a motor pump that circulates air and has a release valve. Thirion et al. [22] introduced a mechanical emulator for validation of BCG data by using a two-motor setup. This system uses a microcontroller to generate a control signal for motor control. The cardiac and respiratory parts are handled by stepper motors. The cardiac signal is emulated through the stepper motor rotation while the breathing signal is emulated by the beam translation using an integrated lead screw.

Pinheiro et al. [23] aimed to develop a model to study the vibrations generated by human heart activity. They developed a simulink model to test their mathematical model and validated against real data of healthy subjects acquired using the BCG sensing system mounted in an office chair and wheelchair.

However, there exist very few systems that have been developed for vibration-based sensors (especially geophone-based systems). Also, they have been validated using a limited amount of real data. To the best of our knowledge, there exists no simulator testbed system that has been validated on a large human dataset (75 subjects). Further, the existing vibration simulator systems use two motors for HR and RR effect, whereas we use only one motor setup.

## 3. Material and Methods

In this section, we describe the simulator testbed design and configuration including the cardiorespiratory signal generation process. Afterwards, we present a brief overview of the vital signs estimation algorithms.

### 3.1. Device Development and Configuration

The hardware design of our system is made up of several components. The main component that acts as the vibration source is a voice coil motor (60VC003). These motors are designed for applications needing only limited displacement and offer precise displacement control. Therefore, they are often used in medical ventilator machines. This motor was selected after extensive experiments and trials with other types of motors.

We use a Raspberry Pi to control the motion of this motor and simulate the cardiorespiratory signal. The control signal is generated from the GPIo pins of a Raspberry Pi (Raspberry Pi Ltd., Cambridge, UK). Afterwards, a 12-bit DAC board (MCP4725, Microchip Technology Inc., Chandler, AZ, USA) is used to convert the digital signal generated from Raspberry Pi to analog by using the I2C communication protocol. This is achieved by using the SDA and SCL pins of the Raspberry Pi. The GPIo pins of Raspberry Pi generate 5 V output that is mapped to 0–4096 levels of 12-bit DAC.

**Figure 2:**
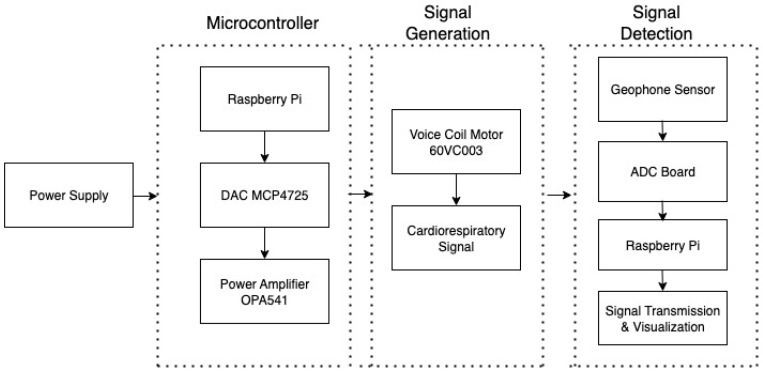
System architecture.

The power output from the Raspberry Pi is not sufficient to drive the motor which requires 24–36 V Operating Voltage. Therefore, a Power Amplifier (OPA541, Texas Instruments, Dallas, TX, USA), powered externally by two ±9 V batteries, is used to amplify the signal to the desired voltage level. The device configuration setup is shown in Figure 1. The system architecture of this design is shown in Figure 2. Next, we describe the sensor data simulation process.

### 3.2. Sensor Data Simulation

For the sensor data simulation, we have developed a Python-based framework that enables the generation of simulated signal from 40 to 240 bpm HR and 8 to 40 bpm RR. This framework uses the Adafruit Circuit Python library to manage the interface of Raspberry Pi and DAC board with the motor. Compared to the existing works that use separate motors to generate HR and RR effects, our framework facilitates the generation of both using a single-motor setup. The simulated signal generation is a multi-step process that is outlined below.

#### 3.2.1. Heartbeat Signal Generation

We start off by experimenting with different types of waves as input to control the motor. For this purpose, sine waves, square waves, Daubechies wavelets, and symlets were tried. In our system, we use a positive cycle wave as an input signal to the motor to control the displacement. The reason behind this is that we need the motor to be hitting ground position to generate a good heartbeat effect. For higher frequencies, the motor is not able to keep up with a full cycle vibration in a short amount of time. The DAC board that we used in our study is 12-bit. For our signal generation, we use an amplitude of 256 as the motor will not hit ground position at higher frequencies due to speed constraints.

**Figure 3:**
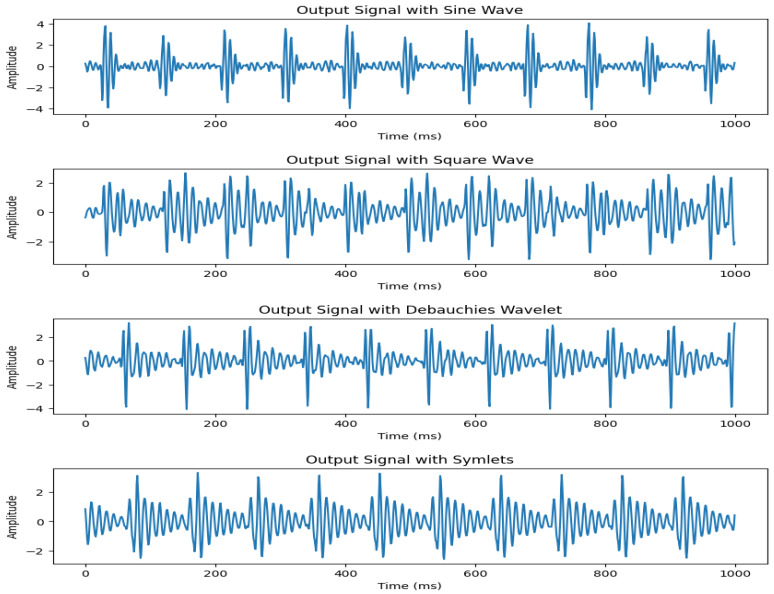
Signal output comparison for sine, square, Daubechies, and symlet waves.

**Figure 4:**
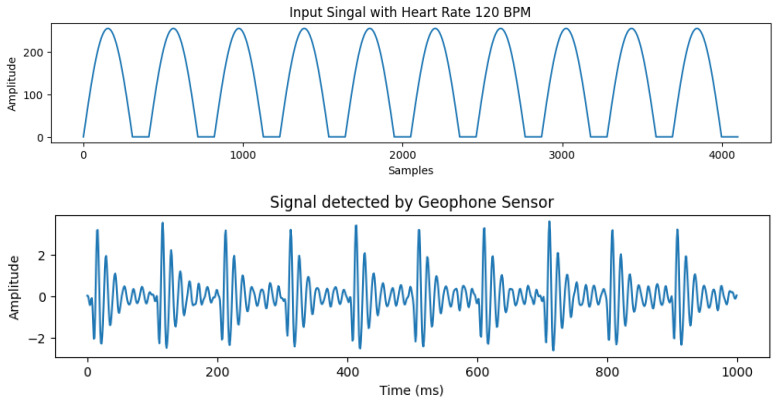
Simulated signal and output.

The experimental results showed that using a sine wave presents the most accurate representation of our required signal with highest correlation to real data. The signal output comparison for different input waves is presented in Figure 3. Therefore, we select sine wave to control the operation of our motor. An example of the input signal with heart rate of 120 bpm and the corresponding output signal detected by the geophone sensor is shown in Figure 4.

#### 3.2.2. Respiration Effect Generation

The generation of the respiration effect from the same motor assembly is not an easy task. Most of the existing works use a separate motor to simulate this effect. However, to make our system compact, we generate the respiration effect from the same motor. This single-source design ensures perfect synchronization between vital signs and reduces mechanical complexity, facilitating the validation of arbitrary HR/RR combinations. We achieve this by modulating the input signal with an envelope that simulates the respiration effect, where the rising slope is proportional to the Respiration Rate (RR).

**Figure 5:**
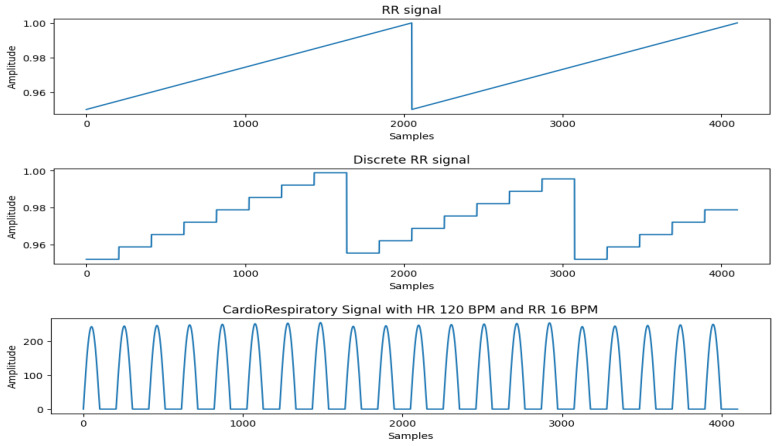
RR signal generation process.

In real-world scenarios, the heart rate (HR) is often not an integer multiple of the respiratory rate (RR), meaning the number of heartbeats per respiratory cycle can vary slightly. To generate the respiration effect and generalize it to arbitrary combinations of HR and RR, we first create a pure respiratory signal in the form of a sawtooth wave. This sawtooth wave is then discretized at the positions where heartbeats occur. The discretized respiratory signal is multiplied with the heart rate signal, applying the respiratory effect to the heartbeats, resulting in a heartbeat signal modulated by respiration. The slope of the sawtooth wave reflects the intensity of respiration and the variation in heartbeat amplitude within a single respiratory cycle. Based on our experiments, we set the minimum value of the sawtooth wave to 0.95 and the maximum to 1. Figure 5 shows the cardiorespiratory signal generated by our system.

### 3.3. Abnormal Heartbeat Generation

The ability to generate abnormal heartbeat such as arrhythmia and other heart rate abnormalities is one of the most important features of our system. In real data collection, it is an extremely difficult and time-consuming process to obtain this kind of data. Our system is able to generate abnormal heartbeat data that can be invaluable for future research. While natural heart rate variability (HRV) is a physiological norm, our system is specifically designed to simulate clinically significant abnormalities. This allows researchers to investigate specific cardiac diseases such as arrhythmia (including Atrial Fibrillation).

**Figure 6:**
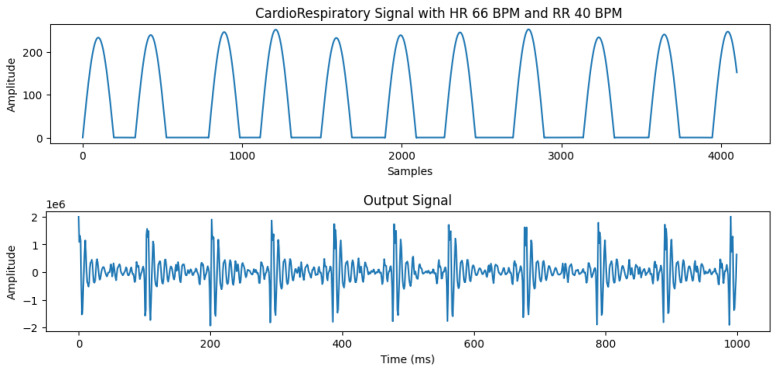
An example of abnormal heartbeat. (**Top**) row shows input signal to the motor while (**bottom**) row shows the signal detected by geophone sensor.

The process to generate abnormal heartbeats involves changing the speed of motor for each heartbeat. This change is user-controlled and can be adjusted to study the effects arising from abnormal heartbeat patterns. An example of simulated abnormal heartbeat that shows irregular patterns such as those observed in Atrial Fibrillation is shown in Figure 6.

### 3.4. Algorithm Design

In this section, we present an overview of the vital signs estimation algorithms. These algorithms have been developed in earlier works [6] and have been shown to work for HR ranges 48–120 bpm and RR ranges 8–25 bpm. Certain improvements to the algorithms are made in order to improve their performance and make them work for higher HR and RR ranges.

#### 3.4.1. Heart Rate Estimation

The estimation of heart rate from the vibration signal is very complicated due to surrounding noise and subject movement. The HR estimation is a multi-step process outlined below:

Step 1: The first step in the vital signs estimation pipeline is data preprocessing. First, we use the z-score normalization to standardize the data. The geophone sensor we used in the study is very sensitive to noise and can pick up vibrations from background emanating from electrical appliances or people walking nearby. To filter out the signal, we use a bandpass filter with range 0.1–20 Hz as this range contains the heartbeat signal information. The low cut-off frequency is selected to remove DC wandering around 0 Hz while the high cut-off frequency essentially removes the high-frequency noise from appliances.

Step 2: For the estimation of heart rate, we use 10 s segments. Typically, the HR is estimated by counting the number of peaks in a signal. However, our signal may not have an accurate amount of peaks in each 10 s duration due to environmental noise. The envelope of the signal is obtained by using Hilbert Transform. This is done by creating an analytic signal and combining the original signal with its Hilbert Transform and calculating the magnitude of the analytic signal to find the envelope. This is given by the following equation:

(1) $$a\left(t\right)=\sqrt{x^{2}\left(t\right)+x_{i}^{2}\left(t\right)}$$

where $x^{2}\left(t\right)$ is the real part and $x_{i}^{2}\left(t\right)$ is the imaginary part of the signal.

Step 3: Next, we use a two-stage moving average filter with a window length 10 followed by 3rd degree polynomial fitting to further smooth and enhance the envelope of the signal obtained in Equation (1). Afterwards, the Auto-Correlation Function (ACF) of the signal is computed. The ACF of the signal gives important information about the periodicity of the signal. The lag-*k* auto-correlation for ${\left\{x_{i}\right\}}_{i=1}^{n}$ is defined as

(2) $$\mathrm{ACF}\left(k\right)=\frac{\sum_{i=1}^{n-k}\left(x_{i}-\bar{x}\right)\left(x_{i+k}-\bar{x}\right)}{\sum_{i=1}^{n}{\left(x_{i}-\bar{x}\right)}^{2}}$$

where $\bar{x}$ is the average value of the signal. Here, the lag-*k* represents the time shift (in samples) applied when computing the auto-correlation of the signal. In our implementation, *k* is not a single fixed value, but is swept over a range of lags corresponding to physiologically plausible heart rate frequencies. The lag corresponding to the dominant ACF peak within the HR range (40–240 bpm) is selected to determine signal periodicity and quality.

Step 4: For accurate HR detection, we set a threshold for the dominant frequency component of ACF to lie between 0.6 and 4 Hz as this contains all the ranges for human HR. This method of using HR has already been proven to be more accurate than estimating the HR from peaks of the raw signal or envelopes. Finally, the HR is given by the following equation:

$$HR=\frac{60}{m}\cdot Fs,$$

where *m* is the mean interval of heartbeats and Fs is the sampling frequency (100 Hz).

#### 3.4.2. Respiratory Rate Estimation

For estimation of RR, we use 50 s segments. The estimation of RR from this signal is very challenging as the RR signal is buried within noise and HR signals. Similar to HR, the RR estimation is a multi-step process summarized below:

Step 1: The geophone sensor generates an electrical signal proportional to the relative velocity between its internal seismic mass and the sensor housing, which corresponds to the velocity of the vibrating surface (body/chest) to which the sensor is coupled. We obtain the chest movement displacement by performing an integral operation on the signal. We can express the output of the geophone sensor as a function of velocity: $x_{i}=C\cdot v_{i}+e_{i}$; here, $x_{i}$ is the output from the geophone, $v_{i}$ is the velocity, and $e_{i}$ is a Gaussian error with mean μ and variance $\sigma^{2}$. By subtracting expectations from both sides, we obtain ${\hat{x}}_{i}=C\cdot {\hat{v}}_{i}+{\hat{e}}_{i}$, with

$${\hat{x}}_{i}=x_{i}-E\left[x_{i}\right],\;{\hat{v}}_{i}=v_{i}-E\left[v_{i}\right],\;\mathrm{and}\;{\hat{e}}_{i}∼N\left(0,\sigma^{2}\right).$$

For RR estimation, we are not concerned with the amplitude of the signal and focus on the waveform. Therefore, *C* can be treated as 1 in this case. This simplifies the relationship to ${\hat{x}}_{i}={\hat{v}}_{i}+{\hat{e}}_{i}$. We propose the following to unveil respiration rate:

(3) $$\begin{array}{c} \;D_{T}=\sum_{i=1}^{T}{\hat{x}}_{i}=\sum_{i=1}^{T}\hat{v_{i}}+\sum_{i=1}^{T}\hat{e_{i}}, \end{array}$$

Finally, substituting $\sum_{i=1}^{T}{\hat{v}}_{i}$ and $\sum_{i=1}^{T}{\hat{e}}_{i}$ with $y_{T}$ and $b_{T}$, respectively, we get

(4) $$D_{T}=y_{T}+b_{T}\;\mathrm{with}\;b_{T}∼N\left(0,T\cdot \sigma^{2}\right),$$

where $D_{T}$ is the displacement at time step *T*.

Step 2: In the above equation, $y_{T}$ contains both the heartbeat and respiration displacements. To extract the respiration signal, we first isolate the low-frequency temporal component of $D_{T}$, which predominantly captures the respiratory motion. This is achieved by computing the envelope of the signal (using Hilbert Transform) and transforming it to the frequency domain using the Fast Fourier Transform (FFT). The resulting spectrum is filtered between 0.1 and 2 Hz to suppress non-respiratory components. Finally, the inverse FFT is applied to obtain the respiration signal in the time domain.

Step 3: We count the peaks on the filtered signal and use the following equation to calculate RR.

$$RR=\frac{60}{duration}\cdot P$$

Here, *P* is the number of peaks and the signal duration of 50 s is used in this study.

## 4. Evaluation

In this section, we present the experiment setup and evaluation of our proposed system.

### 4.1. Experiment Setup

**Figure 7:**
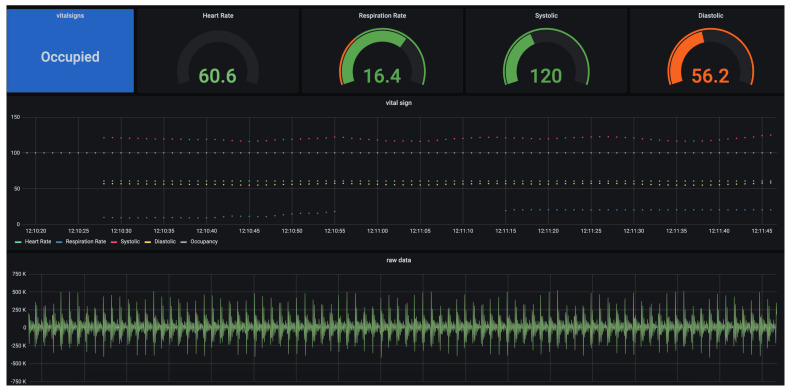
Real-time visualization.

The proposed setup is placed on the bed inside a dummy subject that acts as a human. The primary purpose of this dummy subject is to provide an interface for vibration transmission through a medium rather than mimicking human tissue performance. The geophone sensor/BedDot device [6] is placed underneath and attached using a magnet. This geophone sensor is connected to a separate Raspberry Pi that uses an ADC board to sample the raw data at 100 Hz. The raw data is transmitted to a database and displayed in real time on a Graphical User Interface (GUI) as shown in Figure 7. The specifications of the Raspberry Pi are shown in Table 1.

### 4.2. Qualitative Analysis of Simulated Signal with Real Signal

**Figure 8:**
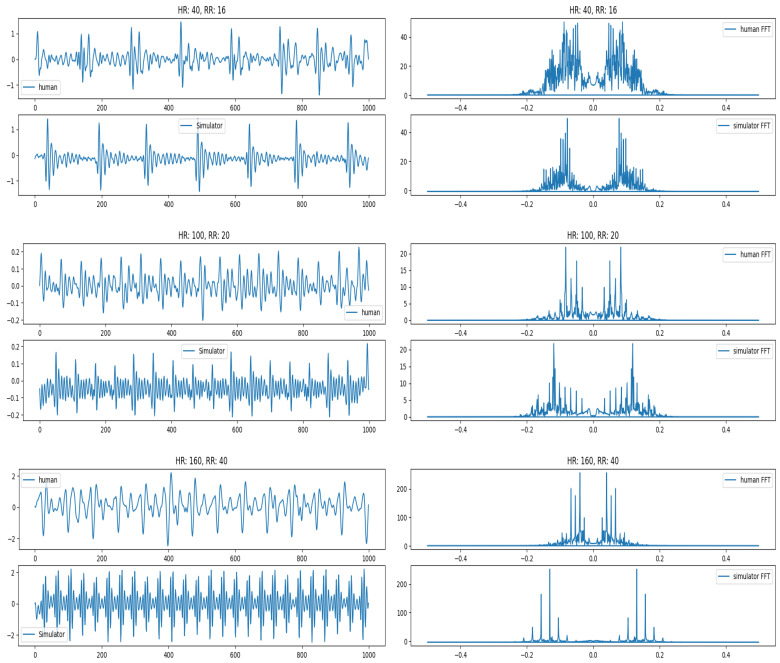
Signal comparison for real and simulated signals at low, medium, and high HR. (**Left**) shows comparison of raw signals with time (in milliseconds) shown on *X*-axis while (**Right**) shows their FFT comparison with normalized frequency shown on *X*-axis.

First, we compare the quality and characteristics of the simulated signal that we generated with the signal obtained from real humans. For this comparison, we used a human dataset comprising 75 people, collected over the last few years. We pick three different points for comparison. They represent lower range (HR: 40, RR: 12), medium range (HR: 100, RR: 20) and high range (HR: 160, RR: 28). We compare the signals in the time and frequency domains. The frequency domain analysis shows that the two signals are similar and the dominant frequency for both real and simulated signals lies within 0.2 normalized frequency (10 Hz) Range. They are shown in Figure 8. It can be seen that at the higher frequencies, the real human signal looks noisier compared with the simulated signal. This is due to the increased weight of humans and is explained later.

**Figure 9:**
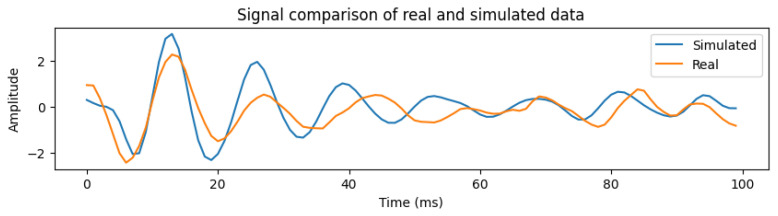
Heartbeat comparison, simulated vs real signal.

Next, we compare heartbeats of real and simulated signals. An example is shown in Figure 9, for HR 60 and RR 20. The two signals show a cross-correlation of 0.85 that signals a strong correlation. While this high coefficient demonstrates strong linear association, temporal alignment, and periodicity between the waveforms, the metric is utilized to validate the consistency of the simulated signals for algorithm stress-testing and development, and may not represent subtle morphological features required for diagnostic clinical decision-making.

An extensive comparison of the simulated and real signals is also done by dividing the signals into 12 HR ranges from 40 to 184 (from different human subjects). This is summarized in Table 2 and Table 3. We calculate the cross-correlation and spectral error between the real and simulated signals. For the cross-correlation computation, the signals are normalized to the same scale and aligned at the lag where they show maximum correlation. The spectral error is defined as Mean Squared Error (MSE) between Power Spectral Density (PSD) values of real and simulated signals. These metrics are computed by extracting a heartbeat cycle from real and simulated data. Afterwards, 20 random samples from each bin are selected and the average metric value for heartbeats is presented. The comparison for higher HR is not available due to unavailability of corresponding human data.

#### 4.2.1. Using Weights to Model Different Humans

**Figure 10:**
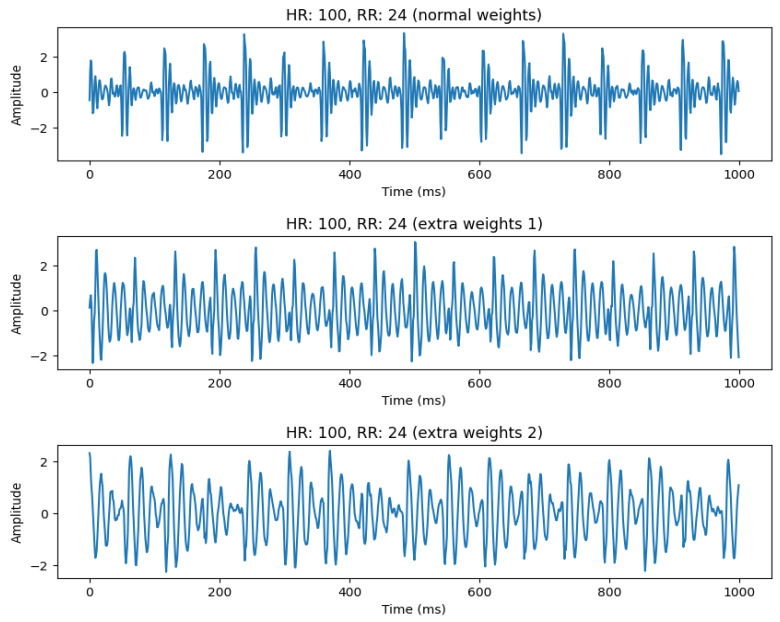
Signal comparison for increasing weights. The (**top**) figure shows the signal when the default weight of the dummy (60 kg) is used. The (**middle**) row shows the signal when 20 kg is added. The (**bottom**) row shows the signal when 40 kg is added.

An important aspect of our system is its ability to model different subjects. This can be achieved by making some hardware modifications to the system, such as adding some extra weights to emulate real subject weights. This is shown in Figure 10, where we emulate three different subjects by adding extra weights to the setup. It can be seen that, with increasing weights, the signal starts to become more distorted. However, the HR and RR can still be estimated with high accuracy, except when the HR becomes very high (>160 bpm). This is discussed in the next section.

#### 4.2.2. Effect of Increasing Weight at High HR

**Figure 11:**
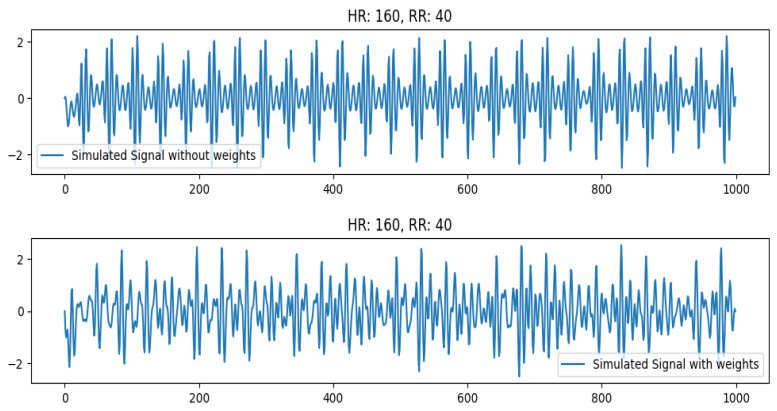
Simulated signal comparison at high HR value (160 bpm) for dummy subject at normal weight (60 kg, shown in (**top**) row) and increased weight (100 kg, shown in (**bottom**) row).

Next, we check the effect of increasing weights at high HR (160 bpm). This is pretty significant as observed in Figure 8. It can be seen that the real signal for HR 160 is slightly different from the simulated signal (with no weights). However, if we add weights to the testbed (to emulate the weight of real humans), the simulated signal becomes distorted and resembles the real human signal of a person with increased weight. The weight of the dummy subject is around 60 kg. In this first case, we add 20 kg to the weight of the dummy. In the second case, 40 kg are added. The signal comparison for these signals is shown in Figure 11. This is due to the vibrations overlapping with each other that may signify a limitation of the sensor to pick up vibrations at very high frequency (>160 bpm) for healthy individuals with more weight. In this case, the prediction accuracy for the signal also drops.

#### 4.2.3. Signal Recovery and Transfer Function Estimation

An important function of our testbed is that it enables us to model the noise and estimate transfer function as the signal changes from source (heart) to sensor under the bed. This is especially significant for highly noisy environments, such as a hospital. The recovery of a clean vibration signal allows the vital signs to be estimated with more accuracy and explore estimation of other vital signs, such as blood pressure, in future research.

The transfer function is estimated by performing an experiment with using a Chirp wave as an input to control the motor motion. The reason behind this is that we aim to model the transfer function of every possible frequency range. In this setup, a geophone-based sensor is placed over the bed and another one is placed underneath. The sensor on the bed represents the clean vibration signal while the second sensor output is highly noisy. The transfer function is estimated by using various signal processing approaches. The results show that using the FFT-based method provides the most accurate signal recovery method. The transfer function is given by

(5) $$H\left(f\right)=\frac{Y(f)}{X(f)}$$

**Figure 12:**
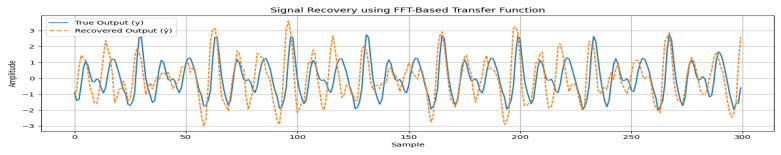
Signal recovery with FFT-based transfer function estimation.

Here, Y(f) represents the output (under the bed), whereas X(f) is the input (over the bed). First, we estimate the transfer function using the above equation, then use the estimated transfer function to recover the original input (signal over the bed). The original and recovered function are shown in Figure 12. It can be observed that the signal can be recovered with high accuracy (90%).

### 4.3. Performance of Algorithm on Simulated and Real Data

In this section, we present a validation of our HR and RR algorithms on simulated and real data. These algorithms are presented in our earlier works [6,7].

#### 4.3.1. Performance on Simulated Data

**Figure 13:**
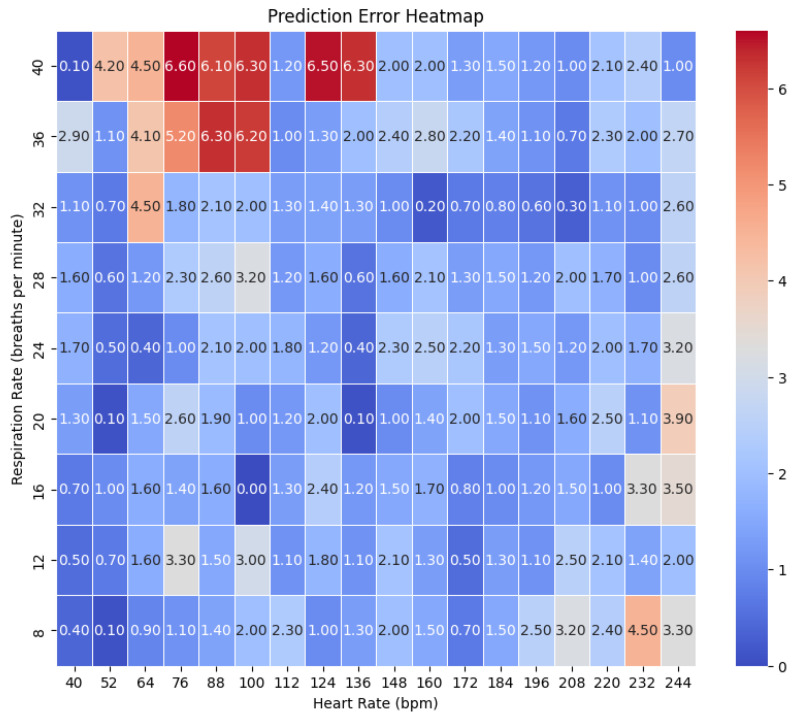
The heatmap of error (in beats per minute) on simulated data for 162 combinations. This represents the maximum error in bpm for the computation of a specific HR/RR combination against the actual HR/RR value.

We generated the simulated data in the testbed assembled in our lab. The system was placed inside a dummy that acts as a human. The setup is shown in Figure 1. We tested around 162 combinations of HR ranges from 40 to 240 bpm and RR from 8 to 40 bpm. The accuracy error heatmaps are presented in Figure 13. They represent the maximum estimation error for an HR/RR prediction against the actual value.

#### 4.3.2. Comparison with Real Data

Next, we compare the performance of these algorithms on the real human and our simulated data. The human dataset used for evaluation consists of data collected from 75 humans over the last few years. Participants’ ages varied around a mean of 50 ± 19 years, consisting of 32 males and 43 females. On average, their heights measured 167 ± 9 cm, with average weights of 81 ± 19 kg. Healthy individuals were recruited that had no significant medical history (cardiac conditions such as arrhythmia). Individuals were asked to lay still for data collection. Any individuals with significant motion changes were excluded from the study. The clinical experiments were approved by IRB PROJECT00001838. The ground labels were collected by using Caretaker4, an FDA-approved device. For the HR evaluation, we segment the data into 10 s segments while for the RR evaluation, we segment the data in 50 s segments. The rationale for window selection is to provide a stable mean interval while remaining responsive to rapid physiological changes. During real-time monitoring, the predictions are made every second based on the previous window data.

The results for both simulated and real human data are summarized in Table 4. The main performance metrics used are Mean Absolute Error (MAE), Mean Absolute Percentage Error (MAPE) and Standard Deviation of Error (STD). The results show that for the human dataset, our model achieves MAE of 1.28 ± 0.48 bpm for HR and 1.46 ± 0.56 bpm for RR. The MAE for simulated data is 1.86 ± 0.20 bpm for HR and 1.87 ± 0.25 bpm for RR. The Mean Absolute Error (MAE) and the Mean Absolute Percentage (MAPE) are defined as follows:

(6) $$\mathrm{MAE}=\frac{1}{n}\sum_{i=1}^{n}\left|{\hat{y}}_{i}-y_{i}\right|$$

(7) $$\mathrm{MAPE}=\frac{100}{n}\sum_{i=1}^{n}\left|\frac{{\hat{y}}_{i}-y_{i}}{y_{i}}\right|$$

where $y_{i}$ is the actual value and ${\hat{y}}_{i}$ is the predicted value.

The Standard Deviation of Error (STD) is defined as follows:

(8) $${\mathrm{STD}}_{\mathrm{error}}=\sqrt{\frac{1}{n-1}\sum_{i=1}^{n}{\left(e_{i}-\bar{e}\right)}^{2}}$$

Here, $e_{i}$ denotes the prediction error for the *i*-th sample, and $\bar{e}$ represents the mean error across all samples.

**Figure 14:**
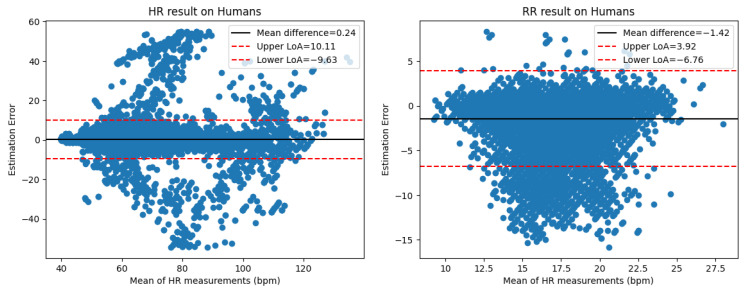
Bland–Altman Plots for HR and RR. The *X*-axis shows the mean of measurements (ground truth and predicted), while the *Y*-axis shows the estimation error.

To assess the agreement between our vital signs monitoring algorithms and the reference standards, we utilized Bland–Altman plots, shown in Figure 14. The plots illustrate the mean difference (bias) and the limits of agreement (±1.96 SD) for both HR and RR.

## 5. Conclusions

In this study, we present SimDot, a simulator testbed for the development and validation of geophone-based contactless vital signs monitoring systems including extreme cases and abnormalities. These systems detect the micro-vibrations caused by the human heart and use the detected signal to estimate vital signs. In our proposed system, a voice coil motor acts as the cardiorespiratory vibration source. We also generate a respiration effect from this single motor, compared to other works that use a separate setup for this purpose. The results show that our system can generate a highly accurate signal from heart rate 40–240 bpm and respiration rate 8–40 bpm. Our system is also able to generate abnormal heartbeat data that is invaluable for the detection of certain heart disorders such as arrhythmia. The generated signal shows a strong correlation (0.85) compared to real human data. The simulated signal is used to develop and improve the vital signs monitoring algorithms with performance metrics showing accuracy (MAE less than 2). Our system is easy to install and will advance the validation of these systems and enable researchers to generate highly accurate data without relying on real data collection that is very cumbersome and time-consuming. In future, we aim to extend the scope of our system by validation on certain age groups (infants/kids, older adults) and explore arrhythmia detection by comparing with real data. We also aim to study other vital signs such as blood pressure. Finally, we intend to extend this system to validate other contactless systems that are based on other vibration-based sensors such as ballistocardiogram (BCG) and seismocardiograph (SCG).

## Figures and Tables

**Table 1:** Raspberry Pi 3 Model B specifications.

CPU	1.2 GHz 64-bit quad-core ARMv8
Memory	1 GB SDRAM
USB 2.0 ports	4 (via the on-board 5-port USB hub)
On-board storage	32 GB Micro SDHC
On-board network	10/100 Mbit/s Ethernet, 802.11 n wireless, redtooth 4.1

**Table 2:** Qualitative comparison of simulated and real signals.

HR Range	Cross-Correlation	STD	HR Range	Cross-Correlation	STD
40–52	0.83	±0.05	113–124	0.85	±0.04
53–64	0.84	±0.04	125–136	0.87	±0.05
65–76	0.83	±0.06	137–148	0.85	±0.04
77–88	0.83	±0.02	149–160	0.84	±0.06
89–100	0.82	±0.03	161–172	0.81	±0.04
101–112	0.86	±0.02	173–184	0.80	±0.09

**Table 3:** Spectral error comparison of simulated and real signals.

HR Range	Spectral Error (MSE)	HR Range	Spectral Error (MSE)
40–52	0.014	113–124	0.031
53–64	0.004	125–136	0.025
65–76	0.021	137–148	0.021
77–88	0.011	149–160	0.033
89–100	0.039	161–172	0.018
101–112	0.022	173–184	0.045

**Table 4:** Summary of results.

Dataset	Vital	MAE	MAPE	STD
Simulated	HR (bpm)	1.86	0.03	1.33
	50 s RR (bpm)	1.87	0.05	1.60
Humans	HR (bpm)	1.28	0.015	2.11
	50 s RR (bpm)	1.46	0.12	2.49

## Data Availability

The data presented in this study are available on request from the corresponding author due to privacy.
